# Direct and Bicarbonate-Induced Iron Deficiency Differently Affect Iron Translocation in Kiwifruit Roots

**DOI:** 10.3390/plants9111578

**Published:** 2020-11-14

**Authors:** Nannan Wang, Xiaoke Dong, Yuanlei Chen, Baiquan Ma, Chunchao Yao, Fengwang Ma, Zhande Liu

**Affiliations:** College of Horticulture, Northwest A&F University, Yangling 712100, China; n.n.wang@nwafu.edu.cn (N.W.); dongxiaoke@nwafu.edu.cn (X.D.); chenyuanlei@nwafu.edu.cn (Y.C.); bqma87@nwsuaf.edu.cn (B.M.); yaocc168@nwafu.edu.cn (C.Y.)

**Keywords:** kiwifruit, iron deficiency, bicarbonate, iron uptake, iron translocation, water-soluble iron

## Abstract

Bicarbonate-induced iron (Fe) deficiency (+Bic) is frequently observed in kiwifruit orchards, but more research attention has been paid to direct Fe deficiency (-Fe) in plants, including kiwifruit. Here we compared the differences of kiwifruit plants between -Fe and +Bic in: (1) the traits of ^57^Fe uptake and translocation within plants, (2) Fe forms in roots, and (3) some acidic ions and metabolites in roots. The concentration of ^57^Fe derived from nutrient solution (^57^Fedfs) in roots was less reduced in +Bic than -Fe treatment, despite similar decrease in shoots of both treatments. +Bic treatment increased ^57^Fedfs distribution in fine roots but decreased it in new leaves and stem, thereby displaying the inhibition of ^57^Fedfs translocation from roots to shoots and from fine roots to xylem of coarse roots. Moreover, +Bic imposition induced the accumulation of water-soluble Fe and apoplastic Fe in roots. However, the opposite was observed in -Fe-treated plants. Additionally, the cell wall Fe and hemicellulose Fe in roots were less reduced by +Bic than -Fe treatment. +Bic treatment also triggered the reduction in H^+^ extrusion and the accumulation of NH_4_^+^, succinic acid, and some amino acids in roots. These results suggest that, contrary to -Fe, +Bic treatment inhibits Fe translocation to shoots by accumulating water-soluble and apoplastic Fe and slowing down the release of hemicellulose Fe in the cell wall in kiwifruit roots, which may be related to the decreased H^+^ extrusion and the imbalance between C and N metabolisms.

## 1. Introduction

Kiwifruit (*Actinidia* Lindl.) is highly susceptible to iron (Fe) deficiency [1], particularly in calcareous soils that comprise approximately 30% of arable land area worldwide [2] and generally contain bicarbonate concentrations in a range of 0.3–4 mM [3,4]. Fe-starved kiwifruit vines frequently exhibit the interveinal chlorosis in young leaves, and the decrease in leaf size and thickness as well as branching rate, and thus a substantial loss of fruit yield and quality [1,5]. More than 30% of global kiwifruit yield is from Shaanxi and Sichuan provinces in China where the orchard soils are characterized by high bicarbonate concentration [6,7]. Consequently, bicarbonate-induced Fe deficiency (+Bic) has become a big challenge for the sustainable development of world kiwifruit industry.

Under direct Fe deficiency (-Fe) conditions, the enhancement of Fe uptake and translocation within plants have been well demonstrated by isotope labeling experiment [8] and analysis of gene expression [9]. Under +Bic conditions, on the contrary, Fe uptake and translocation in plants seems to be blocked. Alhendawi et al., 1997 [3] reported that Fe uptake and translocation declined with increasing bicarbonate concentrations in barley, sorghum, and maize. Later, the inhibition of Fe uptake by roots and its translocation from roots to shoots have been identified by ^59^Fe and ^57^Fe tracing in bicarbonate-treated grape [10] and citrus [11] respectively. Martínez-Cuenca et al., 2013 [12] found that bicarbonate hinders Fe translocation from cotyledons to shoot and root of citrus seedlings. All of the above suggest that bicarbonate hampers Fe uptake and translocation within plants, but the more detailed characteristics in bicarbonate-fed plant tissues, such as Fe translocation between fine and coarse roots or new and old leaves, remain unclear.

Fe forms play vital roles in Fe absorption and translocation within plants [13,14]. Generally, Fe forms can be divided into two Fe pools: water-insoluble Fe, which is mainly bound to cell wall, and water-soluble Fe that is a mixture of apoplastic and intracellular fluids [15,16]. Among them, the cell wall Fe could be further fractionated into pectin Fe, hemicellulose Fe, and cellulose Fe, and most of the cell wall Fe is bound to hemicelluloses [17]. Exogenous application of ABA, NaCl or NH_4_^+^ alleviates Fe deficiency chlorosis by enhancing water-soluble Fe concentration via facilitating the reuse of cell wall Fe (especially of hemicellulose Fe) in plant roots [18,19,20,21]. Moreover, Donnini et al., 2012 [22] observed that apoplastic Fe in roots of *Parietaria diffusa* was increased by bicarbonate treatment, but reduced by low Fe supply. These results imply that Fe forms may function in Fe deficiency responses in plant roots. However, it is still unknown what the differences exist in Fe forms (e.g., water-soluble Fe) of kiwifruit plant roots between -Fe and +Bic conditions.

Numerous ions and metabolites are involved in the responses to Fe deficiency in plants, including proton (H^+^) extrusion, ferric chelate reductase (FCR) activity, NH_4_^+^, NO_3_^-^, and acidic compounds such as organic acids, phenolics, flavins, flavonoids, and polyamines [14,20,23]. In kiwifruit plants, -Fe treatment increased H^+^ extrusion, FCR and phosphoenolpyruvate carboxylase (PEPC) activities in roots but lowered the pH of nutrient solution [24,25,26]. Our recent works indicated that +Bic treatment disrupted ionic balance in both kiwifruit leaves and roots, and resulted in the accumulation of NH_4_^+^ and succinic acid in roots [27,28]. Additionally, amino acid metabolism is crucial for the resistance of various abiotic stresses in plants [29,30]. However, which amino acids in kiwifruit plants are responsive to -Fe or +Bic stress? What discrepancies exist in Fe-deficiency-related ions and metabolites between -Fe and +Bic conditions? These questions are still open.

The objective of this study was to compare the differences between -Fe and +Bic treatments in Fe uptake and translocation within plants as well as Fe forms, acidic ions and metabolites in kiwifruit roots.

## 2. Results

### 2.1. The Traits of ^57^Fe Uptake and Translocation in -Fe and Bicarbonate-Treated Kiwifruit Plants

**Figure 1:**
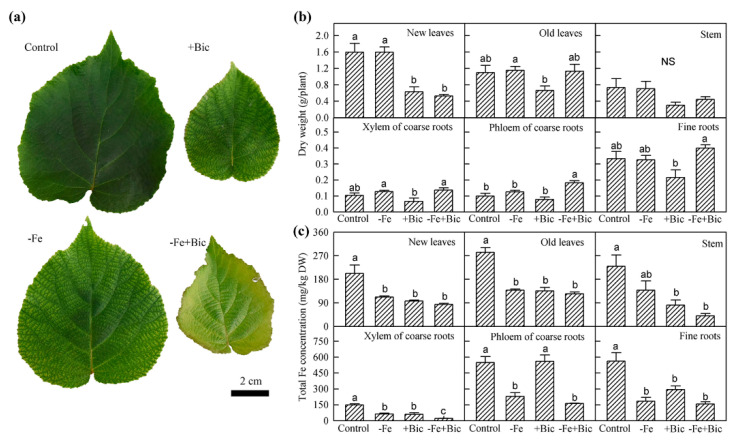
Leaf symptom (**a**), dry weight (**b**) and total Fe concentration (**c**) in various parts of kiwifruit plants treated with direct iron deficiency (-Fe), bicarbonate-induced iron deficiency (+Bic) or both (-Fe+Bic) in solution culture for 28 days. Values are means of three replicates ± standard error (SE). Different letters indicate significant differences among the treatments for the same plant part at *p* < 0.05. DW, dry weight; NS, not significant.

To investigate the traits of Fe uptake and translocation within kiwifruit plants, a stable isotope ^57^Fe-labelling experiment was performed in hydroponics. After 28 days of -Fe and +Bic treatments, typical chlorosis appeared in new leaves and the total Fe concentrations in various plant parts decreased (Figure 1a,c), but the dry weight was not affected except for the reduction in bicarbonate-treated new leaves (Figure 1b). Both -Fe and +Bic treatments decreased the concentration of ^57^Fe derived from nutrient solution (^57^Fedfs) in shoots (including new leaves, old leaves and stem), and the Fe-decreasing effect was further enhanced by combined Fe deficiency (-Fe+Bic) treatment (Table 1). Although the ^57^Fedfs concentrations in xylem and phloem of coarse roots as well as fine roots in -Fe-treated plants decreased by 80.1%, 88.8% and 93.3%, those in +Bic-treated plants decreased by 74.0%, −13.1% and 52.4%, respectively, when compared with the control (Table 1). These results indicate that the ^57^Fedfs concentration in roots was less reduced in +Bic than -Fe treatment, despite similar decrease in shoots of both treatments.

Both -Fe and +Bic treatments decreased ^57^Fedfs distribution in old leaves (Table 1). Moreover, both -Fe and +Bic treatments enhanced ^57^Fedfs translocation rate and relative translocation rate from old leaves to new leaves, even though non statistically significant for +Bic treatment (Table 2). However, both +Bic and -Fe+Bic treatments increased ^57^Fedfs distribution in fine roots but reduced it in new leaves and stem, and the opposite was true for -Fe-treated plants (Table 1). Likewise, both ^57^Fedfs translocation rate and relative translocation rate from roots to shoots in +Bic and -Fe+Bic treatments decreased, although those in -Fe treatment increased (Table 2). Furthermore, a similar trend of ^57^Fedfs translocation from roots to shoots was observed between fine roots and xylem of coarse roots (Table 2). Taken together, contrary to -Fe-treated plants, the main characteristic of +Bic-treated plants is the Fe translocation inhibition from roots to shoots.

### 2.2. Fe Forms in Roots of -Fe and Bicarbonate-Treated Kiwifruit Plants

To unveil the mechanism of Fe translocation inhibition in roots of bicarbonate-treated kiwifruit plants, various Fe forms in roots were analyzed. Water-soluble Fe concentration was unaffected by +Bic treatment, although that was reduced by -Fe and -Fe+Bic treatments (Table 3). Moreover, +Bic treatment increased by 62.1% of apoplastic Fe concentration (Table 3). Considering that the cell wall is one of the most important components of the apoplast, we determined Fe concentration in the cell wall. Both -Fe and +Bic treatments decreased the cell wall Fe, pectin Fe and hemicellulose Fe, but the cell wall Fe was less reduced in +Bic treatment than -Fe and -Fe+Bic treatments (Table 3). Furthermore, the hemicellulose Fe in +Bic-treated plants decreased by 80.1%, while that in -Fe and -Fe+Bic-treated plants decreased by 87.6% and 87.3% in comparison with the control (Table 3). These results suggest that the inhibition of Fe translocation in bicarbonate-treated roots may be associated with the accumulation of water-soluble Fe and apoplastic Fe, and the less reduction in hemicellulose Fe of the cell wall in kiwifruit roots.

### 2.3. Physiological Responses in Roots of -Fe and Bicarbonate-Treated Kiwifruit Plants

**Figure 2:**
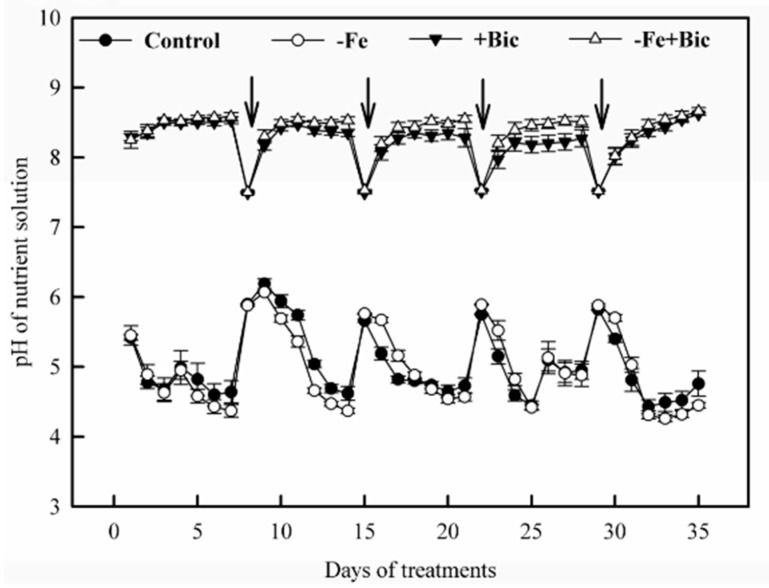
Changes in the pH of nutrient solution for -Fe, +Bic or -Fe+Bic conditions. Values are means of five replicates ± SE. The nutrient solution was renewed every 7 days during the experiment (arrow down).

**Figure 3:**
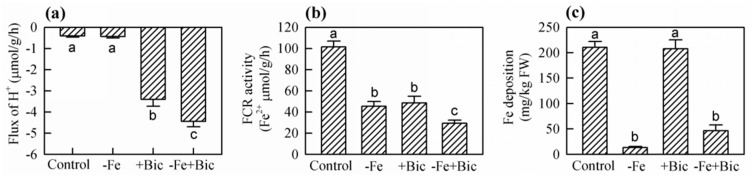
The proton (H^+^) extrusion (**a**), ferric chelate reductase (FCR) activity (**b**) in roots and Fe deposition on root surface (**c**) of kiwifruit plants treated with -Fe, +Bic or -Fe+Bic in solution culture for 35 days. Values are means of five replicates ± SE. Different letters indicate significant differences among the treatments at *p* < 0.05. FW, fresh weight.

To explore the relationship between root Fe translocation inhibition and some physiological parameters related to Fe uptake and transport, we analyzed the nutrient solution pH, H^+^ extrusion, FCR activity and the concentrations of NH_4_^+^, NO_3_^-^, succinic acid, and various amino acids in roots as well as Fe deposition on root surface. When plant roots absorbed ions selectively, the pH of nutrient solution declined in the control and -Fe treatments, but it increased in +Bic and -Fe+Bic treatments (Figure 2). Correspondingly, H^+^ extrusion in +Bic and -Fe+Bic-treated roots decreased (Figure 3a). Both -Fe and +Bic treatments decreased FCR activity in roots, and the effect was more pronounced in -Fe+Bic treatment (Figure 3b). In addition, Fe deposition on the root surface was less in -Fe and -Fe+Bic than the control and +Bic treatments (Figure 3c).

**Figure 4:**
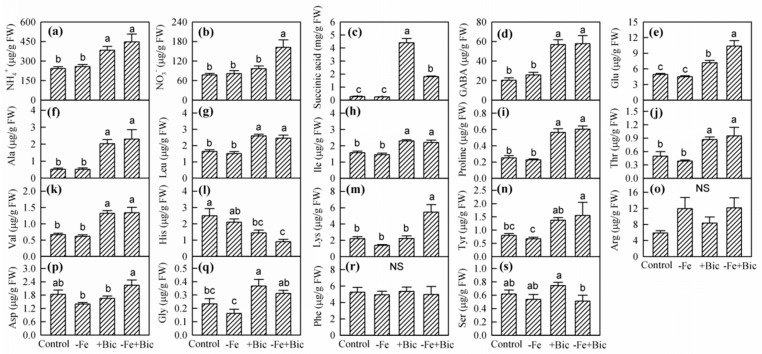
The concentrations of NH_4_^+^ (**a**), NO_3_^-^ (**b**), succinic acid (**c**), and various amino acids (**d**–**s**) in roots of kiwifruit plants treated with -Fe, +Bic or -Fe+Bic in solution culture for 35 days. Values are means of five replicates ± SE. Different letters indicate significant differences among the treatments at *p* < 0.05. FW, fresh weight; NS, not significant. Ala, alanine; Arg, arginine; Asp, aspartic acid; GABA, γ-aminobutyric acid; Glu, glutamic acid; Gly, glycine; His, histidine; Ile, isoleucine; Leu, leucine; Lys, lysine; Phe, phenylalanine; Ser, serine; Thr, threonine; Tyr, tyrosine; Val, valine.

Compared with the control and -Fe treatments, +Bic and -Fe+Bic treatments enhanced the concentrations of NH_4_^+^, succinic acid, GABA, Glu, Ala, Leu, Ile, proline, Thr and Val in roots (Figure 4a,c–k). Moreover, -Fe+Bic treatment increased the concentrations of NO_3_^-^, Lys and Tyr (Figure 4b,m,n). By contrast, +Bic and -Fe+Bic treatments decreased His concentration in roots (Figure 4l). These results suggest that root Fe translocation inhibition might be related to the reduction in H^+^ extrusion and the accumulation of NH_4_^+^ and succinic acid, as well as the metabolic imbalance of some amino acids.

## 3. Discussion

Bicarbonate-induced Fe deficiency is frequently observed in kiwifruit orchards established in calcareous soils [1,31,32]. Our pot experiments showed that both -Fe and +Bic treatments resulted in the reduction in Fe concentration of kiwifruit plant tissues (Figure 1c and Figure S1), which is consistent with previous works [10,33]. Several studies have demonstrated that -Fe treatment enhances Fe uptake and translocation within plants [8,9], but Fe uptake and translocation under +Bic conditions is not fully understood. Using the isotopic Fe tracing technique, Nikolic et al., 2000 [10] and Martínez-Cuenca et al., 2013 [11] revealed that +Bic treatment blocked Fe uptake by roots and its translocation from roots to shoots in grape and citrus respectively. However, the parts in roots or shoots that are affected remain unclear. In the present study, we separated ^57^Fe-fed plants into new leaves, old leaves, stem, xylem and phloem of coarse roots, and fine roots. Our results showed that bicarbonate addition inhibited Fe translocation from roots to shoots (Table 2), which is consistent with previous works [3,10,11]. Moreover, Fe translocation from fine roots to xylem of coarse roots was hampered under +Bic conditions (Table 2), indicating that bicarbonate may hinder axial transport and/or xylem loading of Fe in kiwifruit roots. As for above-ground parts, both -Fe and +Bic treatments enhanced Fe translocation from old leaves to new leaves, even though the enhancing-effect being less evident in +Bic treatment (Table 2), implying that Fe translocation between new and old leaves is not the main difference in -Fe and +Bic treatments. These results suggest that the main traits of bicarbonate-induced Fe deficiency in kiwifruit plants is less related to increased Fe redistribution within leaves, but more to the inhibition of Fe translocation process from roots to shoots, for example, Fe translocation from fine roots to xylem of coarse roots.

Next, we analyzed Fe uptake-related indicators in kiwifruit plants. In +Bic and -Fe+Bic treatments, H^+^ extrusion in roots decreased and correspondingly the pH of nutrient solution increased from 7.51 to 8.45 during each solution cultivation (Figure 2 and Figure 3a), coinciding well with previous works [27,34]. Interestingly, bicarbonate increased the H^+^-ATPase activity and *HA1* expression in kiwifruit [25], citrus [11], *Pisum sativum* [33], and *Medicago ciliaris* [35], especially for bicarbonate-tolerant cultivars. This may be explained by the fact that pH/H^+^ change in bicarbonate-fed roots is a combined consequence of both responses of Fe deficiency and resilience of physiological pH within root tissues. The pH in root tissues is much lower than that in bicarbonate-added solution (Figure 2 and Figure 3a) [36], and thus bicarbonate-treated roots must release much more OH^-^ than H^+^ into growth medium to maintain the normal physiological pH in root tissues. Our results also showed that the solution pH declined but no difference was found between the control and -Fe treatment (Figure 2), probably due to high susceptibility of kiwifruit to Fe deficiency with low reaction of H^+^ extrusion in roots [1,27]. The FCR activity decreased in both -Fe and +Bic treatments (Figure 3b), likely because FCR activity is dependent on Fe supply, solution pH, treatment duration and plant species/cultivar [27]. In addition, Fe deposition on root surface did not differ between the control and +Bic treatments for 35-day cultivation in hydroponics (Figure 3c), which is inconsistent with the results that Fe deposition increased in short-term bicarbonate treatment in kiwifruit (7 days) [27], cucumber (30 min) [37], and rice (15 days) [23], indicating that Fe deposition on root surface is duration-specific.

We further investigated Fe forms. Water-soluble Fe and apoplastic Fe in roots increased by 12.6% and 62.1% in +Bic treatment, but decreased by 72.7% and 28.4% in -Fe treatment (Table 3), which is consistent with the findings reported in *Parietaria diffusa* [22]. Moreover, the cell wall Fe and hemicellulose Fe were less reduced in +Bic than -Fe-treated roots (Table 3). Increasing evidence suggests that alleviation mechanism of Fe chlorosis by exogenous application (e.g., ABA, NaCl or NH_4_^+^) is the enhancement of water-soluble Fe in roots and its translocation to shoots via facilitating the release of cell wall Fe (particularly for hemicellulose Fe) in Fe-deprived roots [18,19,20,21]. Furthermore, the high sensitivity of maize line Mo17 to Fe deficiency is related to low translocation to shoots due to a large pool of Fe being fixed in the form of hemicellulose Fe in root cell wall [17]. Likewise, Fe chlorosis resistance in Cleopatra mandarin is associated with the high Fe accumulation in root apoplast [11]. Therefore, water-soluble Fe (mainly present as extracellular Fe, cytoplasmic Fe and vacuole Fe) and apoplastic Fe (mainly present as extracellular Fe and cell wall Fe) are considered to be available to plants and readily transported to shoots. Interestingly, under +Bic conditions, water-soluble Fe and apoplastic Fe accumulated in kiwifruit roots but their translocation to shoots was blocked (Table 2 and Table 3), probably because +Bic treatment led to the alkalization of root tissues [38], favoring the existence of Fe^3+^ rather than Fe^2+^, thereby resulting in the formation of ferric precipitates in extracellular space and/or the slowdown of Fe transport within cell organelles [37,39]. Taking these results together, Fe translocation to shoots in bicarbonate-treated kiwifruit roots is probably blocked due to (1) a large pool of Fe being fixed in hemicellose of the cell wall and (2) Fe precipitation in extracellular space and/or the slowdown of Fe transport within cell organelles.

Acidic ions and compounds play crucial roles in response to Fe deficiency in plants. Inorganic nitrogen forms are mainly present as NH_4_^+^ and NO_3_^-^, which represent over 70% of the total cations and anions in plants [40], and can influence Fe uptake and translocation within plants through the regulation of cellular pH homeostasis [23,41,42]. Bicarbonate treatment resulted in NH_4_^+^ accumulation in kiwifruit roots (Figure 4a), which was in agreement with our previous study [27]. However, -Fe treatment did not affect NH_4_^+^ concentration in roots (Figure 4a), which is inconsistent with the results obtained in *Arabidopsis thaliana* [20], likely because NH_4_^+^ accumulation is dependent on species/cultivar and treatment duration. The increased NH_4_^+^ triggered glutamic acid (Glu) accumulation by glutamine synthetase and glutamate synthase [43]. Glu is able to synthesize various amino acids and polyamines that enhances the capacity to tolerate Fe deficiency [44] and alkaline stress [45], and to synthesize γ-aminobutyric acid (GABA) via glutamate decarboxylase or via proline [43]. GABA is involved in the synthesis of succinic acid through GABA transaminase and succinic semialdehyde dehydrogenase [46]. Exogenous GABA improves the resistance of apple seedlings to alkaline stress by increasing plant growth, the activities of reactive oxygen species and the concentrations of malate, citric acid and succinic acid [47]. In tricarboxylic acid cycle, oxaloacetic acid derived from succinic acid is responsible for the synthesis of several amino acid, such as Thr, Ile, Leu, Val, and Ala (Figure 4) [43]. The accumulation of these acidic ions and compounds mentioned above in bicarbonate-treated kiwifruit roots may be due to (1) the accumulation of NH_4_^+^ [27] and (2) degradation and synthesis of some protein during amino acid metabolism [29,30]. These results suggest that bicarbonate treatment disrupts the balance between C and N metabolic fluxes in kiwifruit roots.

**Figure 5:**
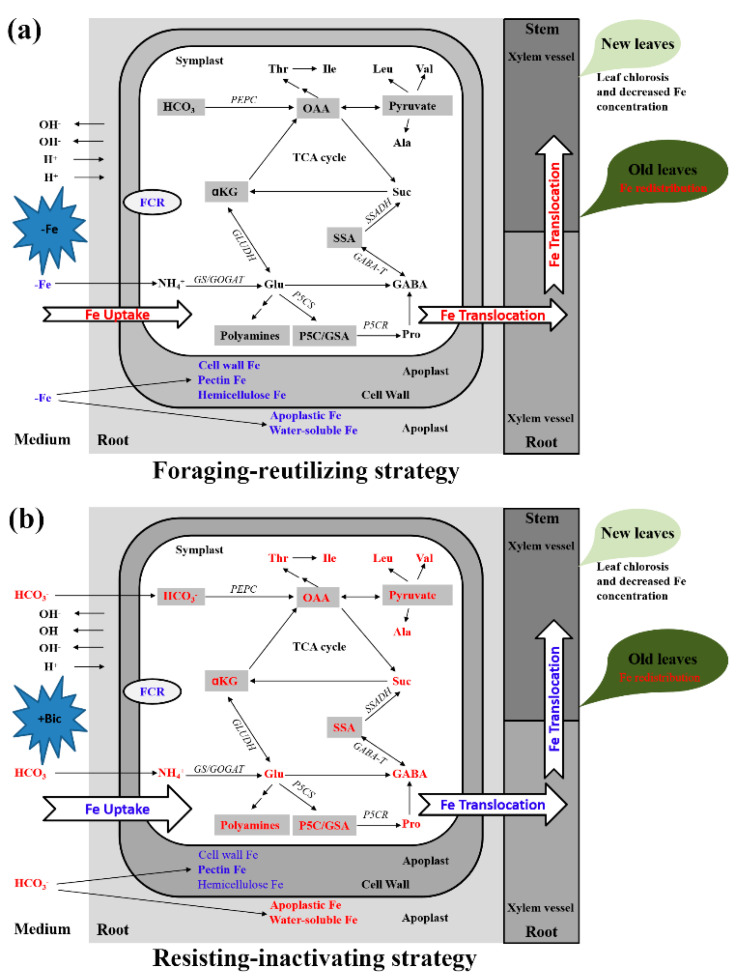
A model for Fe uptake and translocation in kiwifruit plants under direct Fe deficiency (-Fe) (**a**) and bicarbonate-induced Fe deficiency (+Bic) (**b**) conditions. Red and blue-colored parameters or processes represent positive and negative effects, respectively. Gray-shaded parameters in symplast space represent these parameters not identified in the current study. Parameters not in bold indicate these parameters less affected by -Fe and +Bic stresses. The thicker of the arrow representing Fe uptake and translocation, the higher of Fe amount contained. Ala, alanine; αKG, α-ketoglutarate; FCR, ferric chelate reductase; GABA, γ-aminobutyric acid; GABA-T, γ-aminobutyric acid transaminase; Glu, glutamic acid; GLUDH, glutamate dehydrogenase; GS/GOGAT, glutamine synthetase/glutamate synthase; Ile, isoleucine; Leu, leucine; OAA, oxaloacetic acid; P5C/GSA, pyrroline-5-carboxylate/glutamate-semialdehyde; P5CR, pyrroline-5-carboxylate reductase; P5CS, pyrroline-5-carboxylate synthase; PEPC, phosphoenol pyruvate carboxylase; Pro, proline; SSA, succinic semialdehyde; SSADH, succinic semialdehyde dehydrogenase; Suc, succinic acid; TCA, tricarboxylic acid; Thr, threonine; Val, valine.

Based on our current results and previous related works, here we propose a model for Fe uptake and translocation in ‘Qihong’ kiwifruit treated with -Fe or +Bic (Figure 5). Under -Fe conditions, a foraging-reusing strategy is employed by roots to increase Fe absorption from low Fe-supplied nutrient solution, to accelerate the release of hemicellulose Fe from the cell wall and the redistribution of water-soluble Fe and apoplastic Fe in roots as well as total Fe in old leaves, thereby enhancing Fe uptake and translocation within plants, and thus alleviating severe Fe starvation of shoots and new leaf chlorosis. Under +Bic conditions, a resisting-inactivating strategy is used by roots to slow down Fe absorption from bicarbonate-added sufficient Fe solution due to high bicarbonate-induced Fe inactivation, to mitigate the acceleration of the release of hemicellulose Fe from the cell wall and to accumulate water-soluble Fe and apoplastic Fe in alkalized roots, and to minimize the enhancement of Fe redistribution in old leaves, thereby inhibiting Fe uptake and tranlocation within plants, thus causing mild Fe starvation of shoots and new leaf chlorosis.

## 4. Materials and Methods

### 4.1. Plant Material and Treatments

Tissue-cultured plants of kiwifruit cultivar ‘Qihong’ (*Actinidia chinensis* var. *chinensis* ‘Qihong’), sensitive to bicarbonate stress [48], were used in this study. Two hydroponic experiments were performed. Prior to the experiments, the plants were grown in black plastic pots (8 cm × 8 cm × 8 cm; one plant per pot) filled with peat, perlite and vermiculite (5:1:1, *v/v/v*) medium.

In the first experiment, four treatments were established: (1) control, with 25 µM ^57^Fe-EDTA in nutrient solution; (2) direct Fe deficiency (-Fe), with 1.25 µM ^57^Fe-EDTA in nutrient solution; (3) bicarbonate-induced Fe deficiency (+Bic), with 25 µM ^57^Fe-EDTA, 0.25 g/L CaCO_3_ and 0.42 g/L NaHCO_3_ in nutrient solution; and (4) combined Fe deficiency (-Fe+Bic), with 1.25 µM ^57^Fe-EDTA, 0.25 g/L CaCO_3_ and 0.42 g/L NaHCO_3_ in nutrient solution. ^57^Fe-EDTA was prepared by the following procedure [49]: enriched ^57^Fe_2_O_3_ (95.47% isotopic enrichment; Isoflex, San Francisco, CA, USA) was firstly dissolved by concentrated HCl, and then slowly added into the ligand solution (^57^Fe:EDTA molar ratio of 1:1.1). Thereafter, the solution was left overnight, filtered through a membrane filter (0.45 µm pore size) and adjusted to a final volume for ^57^Fe-EDTA stock solution. On 6 September 2017, the plants with 3–5 leaves were transferred into nutrient solution in a growth chamber with a day/night regime of 14 h/10 h light at a photosynthetic photon flux density of 150 µM/m^2^/s, and 24 °C/18 °C temperature, and 70% relative humidity. The nutrient composition for hydroponic and the plant preculture followed our previous work [27]. The nutrient solution was continuously aerated and renewed every 7 days. Each experimental treatment was replicated three times and 15 plants per container (30 cm × 22 cm × 10 cm) served as one replication. The experiment was conducted for 28 days until leaf chlorosis appeared in the apical leaves of Fe deficiency vines. At 9:00–11:00 a.m. on 5 November 2017, new leaves (emerged since the beginning of the treatments), old leaves (emerged before the treatments), stem, xylem of coarse roots (≧2 mm), phloem of coarse roots, and fine roots (<2 mm) were sampled separately. The roots rinsed once with 0.05 M HCl for 3 min to remove extracellular Fe physically bond to root surface and then all the plant parts were rinsed twice with deionized water. The samples were then quickly blotted with tissue paper, oven dried at 65 °C for 72 h, weighed and ground for the total Fe and ^57^Fe determination.

In the second experiment, four treatments were the same as ^57^Fe-labeling experiment, except for natural Fe-EDTA instead of ^57^Fe-EDTA. On 21 April 2018, the plants with 5–7 leaves were grown in hydroponics under glasshouse conditions (natural sunlight, 23–28 °C/15–20 °C day/night temperatures, and 80% relative humidity), at the Agriculture Experiment Station (34°18’ N, 108°4’ E, 521 masl), Northwest Agriculture & Forestry University, Yangling, China. Considering that the plants used in this experiment were larger than those in the ^57^Fe experiment (plants of 5–7 leaves vs. 3–5 leaves), the period of this experiment was prolonged to 35 days and the concentration of -Fe treatment was changed from 1.25 µM to 0.25 µM. Each treatment had five replicates and 24 plants per container (42 cm × 30 cm × 12 cm) served as one replication. The pH of the nutrient solution was monitored daily. At 9:00–11:00 a.m. on 3 July 2018, new leaves, old leaves, stem, xylem and phloem of coarse roots, and fine roots were sampled separately for the measurement of dry weight and total Fe concentration. The fresh fine roots were sampled and stored at −70 °C for the following analysis.

### 4.2. Determination of Total Fe and ^57^Fe Concentration

Approximately 0.30 g of each powdered sample was dry ashed in a muffle furnace at 520 °C for 8 h, dissolved in 0.25 M HNO_3_ to a final volume of 30 mL. One aliquot of the extraction was used to measure total Fe concentration with inductively coupled plasma optical emission spectrometry (ICP-OES). The other aliquot was used to determine ^57^Fe enrichment using inductively coupled plasma mass spectrometry (ICP-MS, Agilent 7700x; Agilent Technologies, Manchester, UK) [49]. The ^57^Fe and ^56^Fe concentrations in plant parts were quantified by an enriched ^57^Fe standard solution (MS57FE-10PPM-100ML; Inorganic Ventures, Christiansburg, VA, USA) and an enriched ^56^Fe standard solution (163.61(38)·10^−6^ mol (^56^Fe)·kg^−1^ (solution); Spike Isotopic Reference Material IRMM-634, B-2440 GEEL, Belgium). The concentration of ^57^Fe derived from nutrient solution (^57^Fedfs) was calculated by subtracting the natural abundance of ^57^Fe in each plant tissue from the abundance determined in the corresponding tissue after the labelling experiment through the following equation:

^57^Fedfs concentration = ^57^Fe in sample − [^56^Fe in sample/(^56^Fe/^57^Fe) in background] (1)

where ^57^Fe in sample is the ^57^Fe concentration in different plant parts, ^56^Fe in sample is the ^56^Fe concentration in different plant parts, (^56^Fe/^57^Fe) in background is 42.28 ± 0.04 based on analysis of three nonenriched kiwifruit leaf samples prior to the labeling experiment.

^57^Fedfs distribution was expressed as the percentage of newly absorbed ^57^Fedfs content (concentration × dry weight) in one plant part to the total newly absorbed ^57^Fedfs content in the whole plant. The translocation rate represents the ratio between ^57^Fedfs distribution in various plant parts, and the relative translocation rate represents the ratio between ^57^Fedfs concentration in various plant parts.

### 4.3. Measurement of Various Fe Forms

Water-soluble Fe was extracted according to the protocol described by Zhu et al. [19]. About 0.80-g fresh root sample was ground in liquid nitrogen and extracted with 8 mL deionized water at room temperature. After centrifugation at 12,000 rpm for 10 min, the supernatant was collected for the measurement of water-soluble Fe concentration using ICP-OES.

Apoplastic Fe was analyzed following the method of Jin et al. [50]. Roots were placed into beakers with 0.5 mM CaSO_4_ under vigorous aeration for 15 min. Then, the roots were transferred to 10 mL of 1.5 mM 2.2-bipyridyl, and the solution was bubbled with N_2_ for 5 min to displace dissolved O_2_ and added with 12.5 mM sodium dithionite (Na_2_S_2_O_4_) for another 5 min to reduce Fe^3+^ to Fe^2+^. Finally, the concentration of Fe^2+^-bipyridyl complex in the solution was measured spectrophotometrically at 520 nm to calculate the amount of apoplastic Fe released by roots.

Cell wall extraction and fractionation were performed as described by Zhu et al. [19]. The pellet after water-soluble Fe extraction was combined with 8 mL of 75% ethanol for 20 min. After centrifugation at 12,000 rpm for 10 min, the pellet was further washed with 5 mL acetone, 5 mL 1:1 (*v/v*) methanol/chloroform, and 5 mL methanol for 20 min respectively. The residual was the faction of the cell wall and dried at 60 °C for the further analysis. The cell wall Fe was determined as the same method of total Fe. Pectin was extracted from the pellets of cell wall via three times of 1-h incubation in hot water, and then hemicellulose was obtained from the pectin-extracted pellets via two times of 12-h incubation in 24% KOH. The extracts were used for the measurement of pectin Fe and hemicellulose Fe using ICP-OES.

### 4.4. Assay of Ferric Chelate Reductase (FCR) Activity, Proton (H^+^) Extrusion in Roots and Fe Deposition on Root Surface

The assay of FCR activity and H^+^ extrusion in roots and Fe plaques on root surface was well described by Wang et al. [27]. For the measurement of FCR activity, 0.25 g root segments were rinsed with 0.2 mM CaSO_4_ for 5 min. Thereafter, the roots were placed into 5 mL of pH 5.5 solution containing 0.5 mM CaSO_4_, 0.1 mM 4-morpholineethanesulfonic acid (MES), 0.1 mM bathophenanthroline-disulfonic acid disodium salt hydrate (Na_2_-BPDS), and 0.1 mM Fe^3+^-ethylenediaminetetraacetic acid (Fe^3+^-EDTA). The roots were then incubated for 1 h in an orbital shaker at 25 °C in the dark and the FCR activity in the solution was determined spectrophotometrically at 535 nm. For the assay of H^+^ extrusion, 0.30 g roots were incubated in a 5 mL of pH 6.2 solution containing 10 mM KCl and 1 mM CaCl_2_ for 3 h and then back titrated with 1 mM HCl or 1 mM NaOH. For the determination of root iron plaques, the roots were incubated in 30 mL of pH 6.5 solution for 3 h. The solution contained 0.27 M sodium citrate (Na_3_C_6_H_5_O_7_·2H_2_O), 0.11 M NaHCO_3_ and 3.0 g Na_2_S_2_O_4_. The filtrate was analyzed for Fe concentration with ICP-OES.

### 4.5. Detection of NH_4_^+^ and NO_3_^-^ Concentrations

The NH_4_^+^ and NO_3_^-^ concentrations in roots were measured as described previously [27]. For NH_4_^+^ determination, a 0.1-g sample was powdered in liquid nitrogen and extracted with 1 mL of 0.1 M HCl and 500 μL chloroform at 4 °C for 15 min. The solution was centrifuged at 12,000 rpm at 4 °C for 10 min, and the supernatant was transferred to a tube with 50 mg activated charcoal, mixed thoroughly, and centrifuged at 12,000 rpm at 4 °C for 5 min. About 100 μL supernatant was firstly well mixed with 500 μL solution containing 1% (*v/v*) phenol and 0.005% (*w/v*) sodium nitroprusside and then mixed with 500 μL solution containing 1% (*w/v*) sodium hypochlorite and 0.5% (*w/v*) NaOH. Thereafter, the solution was incubated in a 37 °C water bath for 30 min and the NH_4_^+^ concentration was analyzed spectrophotometrically at 620 nm. For NO_3_^-^ analysis, a 0.1-g fresh sample was incubated in 1 mL deionized water at 45 °C for 1 h, centrifuged at 6000 rpm at 20 °C for 15 min. The supernatant (0.2 mL) was initially thoroughly mixed with 0.8 mL of 5% (*w/v*) salicylic acid in concentrated H_2_SO_4_, and then added 19 mL of 2 M NaOH to raise the solution pH above 12. Thereafter, the solution was incubated at room temperature for 20 min, and allowed to cool to room temperature. The NO_3_^-^ concentration was detected spectrophotometrically at 410 nm.

### 4.6. Analysis of Succinic Acid and Various Amino Acids

Organic acids in roots were extracted as described previously [27]. Briefly, a 0.15-g sample was powdered in liquid nitrogen, extracted with 1.5 mL deionized water for 30 min, centrifuged at 8000 rpm at 4 °C for 10 min. The supernatant was passed through a 0.22-µm syringe filter into a vial for organic acid analysis using a high-performance liquid chromatography (HPLC) with an Athena C18 column (100 Å, 4.6 × 250 mm, 5 µm) [51].

The concentrations of various amino acids in roots were determined by the method proposed by Zhang et al. [52]. Approximately 0.5-g sample was homogenized with 1 mL solution containing 50% ethanol and 0.1 M HCl, and centrifuged at 12,000 rpm at 4 °C for 10 min. The supernatant was used for the measurement of various amino acids using a liquid chromatography-mass spectrometry (LC-MS, LC: AC, ExionLC; MS: Q-trap5500, AB Sciex).

### 4.7. Statistical Analysis

All the data were presented as the means ± standard errors (SEs) with three or five replicates. The treatment effect was separated by Duncan’s test at *p* < 0.05 using SPSS for Windows 16.0 (SPSS Inc., Chicago, IL, USA).

## 5. Conclusions

Our results indicated that the Fe concentration in kiwifruit roots was less reduced in +Bic than -Fe treatment, despite similar reduction in Fe concentration of shoots for both treatments. Contrary to -Fe, +Bic blocked Fe translocation from roots to shoots and from fine roots to xylem of coarse roots. The inhibition of root Fe translocation in +Bic-treated plants was a consequence of the accumulation of water-soluble Fe and apoplastic Fe, as well as the smaller reduction in hemicellulose Fe of the cell wall. Addition, Fe translocation inhibition may be associated with the decreased H^+^ extrusion and the imbalance between C and N metabolisms. These results shed light on the molecular mechanisms of bicarbonate-induced Fe deficiency in kiwifruit vines, which often occurs in calcareous soils.

## Figures and Tables

**Table 1:** The concentration and distribution of ^57^Fe derived from nutrient solution (^57^Fedfs) in new leaves, old leaves, stem, xylem of coarse roots, phloem of coarse roots, and fine roots of kiwifruit plants treated with -Fe, +Bic or -Fe+Bic in solution culture for 28 days. Values are means of three replicates ± SE. Different letters indicate significant differences among the treatments for the same plant part at *p* < 0.05. DW, dry weight.

Treatment	New Leaves	Old Leaves	Stem	Xylem of Coarse Roots	Phloem of Coarse Roots	Fine Roots
**^57^Fedfs Concentration (mg/kg DW)**						
Control	141.2 ± 29.4a	132.5 ± 23.3a	154.6 ± 24.2a	95.5 ± 12.5a	216.9 ± 24.6a	398.8 ± 66.3a
-Fe	36.9 ± 2.9b	14.7 ± 5.3b	52.3 ± 15.3b	19.0 ± 2.8bc	24.4 ± 3.3b	26.9 ± 6.9c
+Bic	44.6 ± 6.3b	16.8 ± 2.7b	39.5 ± 9.1b	24.8 ± 2.4b	245.4 ± 29.8a	189.7 ± 28.8b
-Fe+Bic	0.9 ± 0.5b	0.7 ± 0.2b	1.3 ± 0.2b	2.0 ± 0.1c	12.8 ± 0.3b	31.8 ± 2.0c
**^57^Fedfs Distribution (%)**						
Control	34.8 ± 3.1b	22.5 ± 0.9a	16.9 ± 2.2b	1.6 ± 0.2ab	3.4 ± 0.2b	20.9 ± 1.2c
-Fe	49.1 ± 4.9a	13.4 ± 3.6b	26.3 ± 1.1a	2.0 ± 0.1a	2.5 ± 0.1b	6.8 ± 0.7d
+Bic	25.0 ± 0.4c	10.1 ± 1.7bc	9.9 ± 1.2c	1.4 ± 0.1b	17.3 ± 2.5a	36.4 ± 3.3b
-Fe+Bic	2.6 ± 1.1d	4.5 ± 1.6c	3.3 ± 0.5d	1.6 ± 0.1ab	13.8 ± 1.0a	74.3 ± 2.2a

**Table 2:** ^57^Fedfs translocation differences in various parts of kiwifruit plants treated with -Fe, +Bic or -Fe+Bic in solution culture for 28 days. Values are means of three replicates ± SE. Different letters indicate significant differences among the treatments at *p* < 0.05. NL, new leaves; OL, old leaves; S, shoots; R, roots; XCR, xylem of coarse roots; FR, fine roots.

Treatment	^57^Fedfs Translocation Rate			^57^Fedfs Relative Translocation Rate		
	NL/OL	S/R	XCR/FR	NL/OL	S/R	XCR/FR
Control	1.05 ± 0.06b	0.46 ± 0.05b	0.25 ± 0.04b	1.56 ± 0.16b	2.88 ± 0.20b	0.08 ± 0.01b
-Fe	3.10 ± 0.93a	1.34 ± 0.07a	0.75 ± 0.10a	4.48 ± 1.54a	7.97 ± 0.67a	0.29 ± 0.02a
+Bic	2.71 ± 0.31ab	0.19 ± 0.03c	0.14 ± 0.03b	2.60 ± 0.41ab	0.82 ± 0.07c	0.04 ± 0.01bc
-Fe+Bic	1.20 ± 0.25b	0.04 ± 0.01d	0.06 ± 0.00b	0.56 ± 0.10b	0.12 ± 0.04c	0.02 ± 0.00c

**Table 3:** The concentrations of water-soluble Fe, apoplastic Fe, cell wall Fe, pectin Fe, and hemicellulose Fe in roots of kiwifruit plants treated with -Fe, +Bic or -Fe+Bic in solution culture for 35 days. Values are means of five replicates ± SE. Different letters indicate significant differences among the treatments for each given parameter at *p* < 0.05. Values inside parentheses represent the percent change (%) in treated plants when compared with the control ones. FW, fresh weight.

	Water-Soluble Fe (mg/kg FW)	Apoplastic Fe (Fe^2+^ nmol/g/h)	Cell Wall Fe (mg/kg Cell Wall)	Pectin Fe (mg/kg Cell Wall)	Hemicellulose Fe (mg/kg Cell Wall)
Control	1.7 ± 0.3a	28.3 ± 1.4b	3907.5 ± 300.4a	490.2 ± 103.7a	358.9 ± 45.1a
-Fe	0.5 ± 0.1b	20.3 ± 2.7b	572.5 ± 42.1c	5.9 ± 0.6b	44.7 ± 5.2b
	(−72.7)	(−28.4)	(−85.3)	(−98.8)	(−87.6)
+Bic	2.0 ± 0.2a	46.0 ± 4.5a	1186.2 ± 115.4b	2.1 ± 0.1b	71.3 ± 8.5b
	(+12.6)	(+62.1)	(−69.6)	(−99.6)	(−80.1)
-Fe+Bic	0.3 ± 0.1b	29.6 ± 6.6b	527.6 ± 49.5c	1.6 ± 0.7b	45.5 ± 5.9b
	(−80.9)	(+4.6)	(−86.5)	(−99.7)	(−87.3)

## Supplementary Materials

The following are available online at https://www.mdpi.com/2223-7747/9/11/1578/s1, Figure S1: The dry weight (a) and total Fe concentration (b) in various parts of kiwifruit plants treated with -Fe, +Bic or -Fe+Bic in solution culture for 35 days. Values are means of five replicates ± SE. Different letters indicate significant differences among the treatments for the same plant part at *p*<0.05. DW, dry weight; NS, not significant.
