# Supplementary material for: Direct and Bicarbonate-Induced Iron Deficiency Differently Affect Iron Translocation in Kiwifruit Roots
Source: Plants (Basel). 2020 Nov 14;9(11):1578. doi: 10.3390/plants9111578 (PMC7696116; doi:10.3390/plants9111578)
Supplement: Supplementary file 1 [file plants-09-01578-s001.pdf]

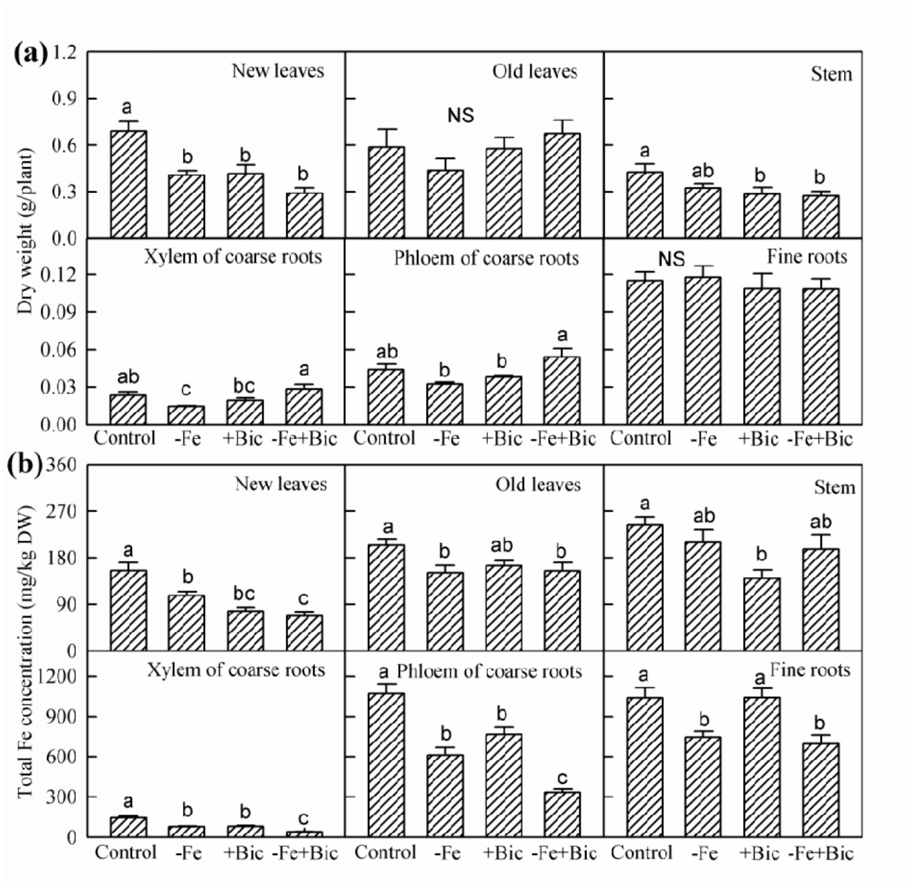

Figure S1. The dry weight (**a**) and total Fe concentration (**b**) in various parts of kiwifruit plants treated with -Fe, +Bic or -Fe+Bic in solution culture for 35 days. Values are means of five replicates ± SE. Different letters indicate significant differences among the treatments for the same plant part at $p < 0.05$. DW, dry weight; NS, not significant.
